# Supplementary figures and images for: Phase variation of Clostridioides difficile colony morphology occurs via modulation of cell division
Source: PLoS Pathog. 2025 Dec 1;21(12):e1013471. doi: 10.1371/journal.ppat.1013471 (PMC12694794; doi:10.1371/journal.ppat.1013471)

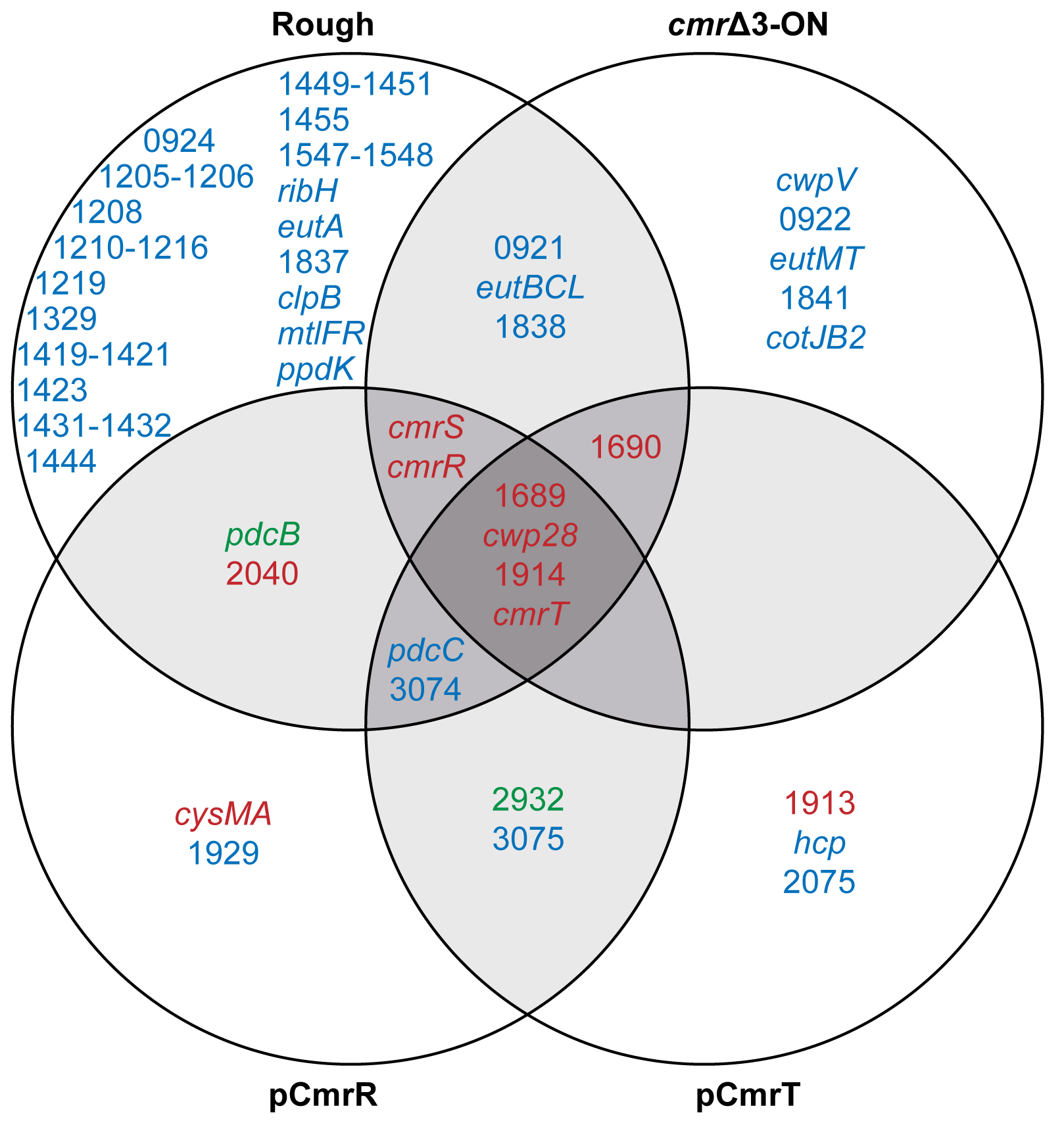

Supplement: S1 Fig — Venn diagram of differentially expressed genes (DEGs) as determined by RNA-Seq analysis. Genes in the circle labeled “Rough” were differentially expressed in the Rough vs. Smooth RNA-Seq comparison. Similarly, “cmrΔ3-ON” contains DEGs from the cmrΔ3-ON vs. -OFF comparison, “pCmrR” contains DEGs from the pCmrR vs. vector comparison, and “pCmrT” contains DEGs from the pCmrT vs. vector comparison. Genes in blue text had increased transcript abundance and genes in red text had decreased transcript abundance in the above comparisons. Green text indicates genes that appeared to be regulated in opposite directions (e.g., higher transcript abundance in one comparison and decreased transcript abundance in the other). (TIF) [file ppat.1013471.s001.tif]

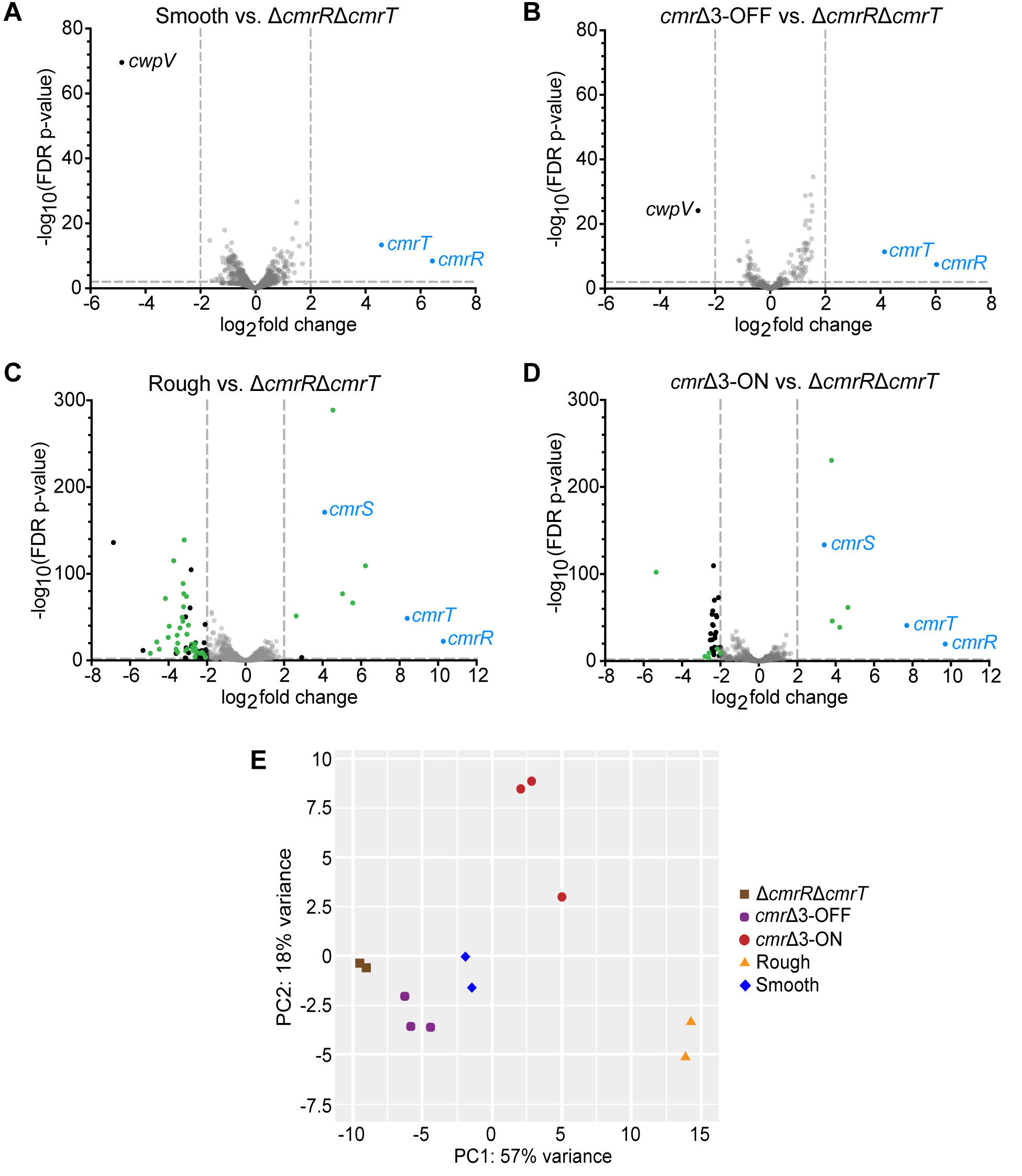

Supplement: S2 Fig — (A-D) Volcano plots of differences in transcript abundance in strains grown on BHIS-agar for 24 hours and collected for RNA-Seq analysis. Gray dotted lines demarcate cutoffs of > 2 log2 fold-change and/or FDR p-value < 0.01. Black dots are genes that met cutoffs, gray dots indicate genes that did not meet the cutoffs, and blue dots denote cmrRST. (A) Wildtype smooth versus ΔcmrRΔcmrT colonies. (B) cmrΔ3-OFF versus ΔcmrRΔcmrT colonies. (C) Wildtype rough versus ΔcmrRΔcmrT colonies. Green dots are genes that met cutoffs and were also differentially expressed in the rough vs. smooth comparison (Fig 1C and Table 1). (D) cmrΔ3-ON versus ΔcmrRΔcmrT colonies. Green dots are genes that met cutoffs and were also differentially expressed in the cmrΔ3-ON vs. cmrΔ3-OFF comparison (Fig 1D and S3 Table). (E) A principal component analysis plot showing clustering of the wildtype rough, wildtype smooth, cmrΔ3-ON, cmrΔ3-OFF, and ΔcmrRΔcmrT colony transcriptomes (n = 2–3). Plot was generated via DESeq2 with regularized logarithm transformed data. (TIF) [file ppat.1013471.s002.tif]

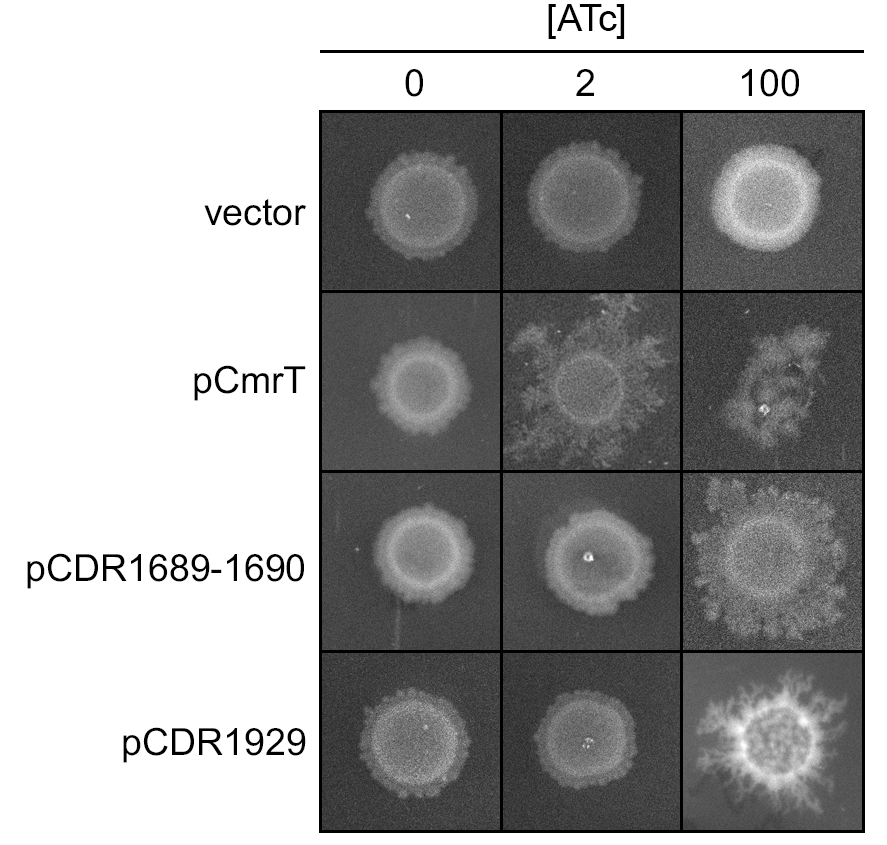

Supplement: S3 Fig — Surface motility plates measured in Fig 4 were imaged with a Syngene G:Box after seven days at 37°C. Shown are representative images from three experiments. Images are not to scale. (TIF) [file ppat.1013471.s003.tif]

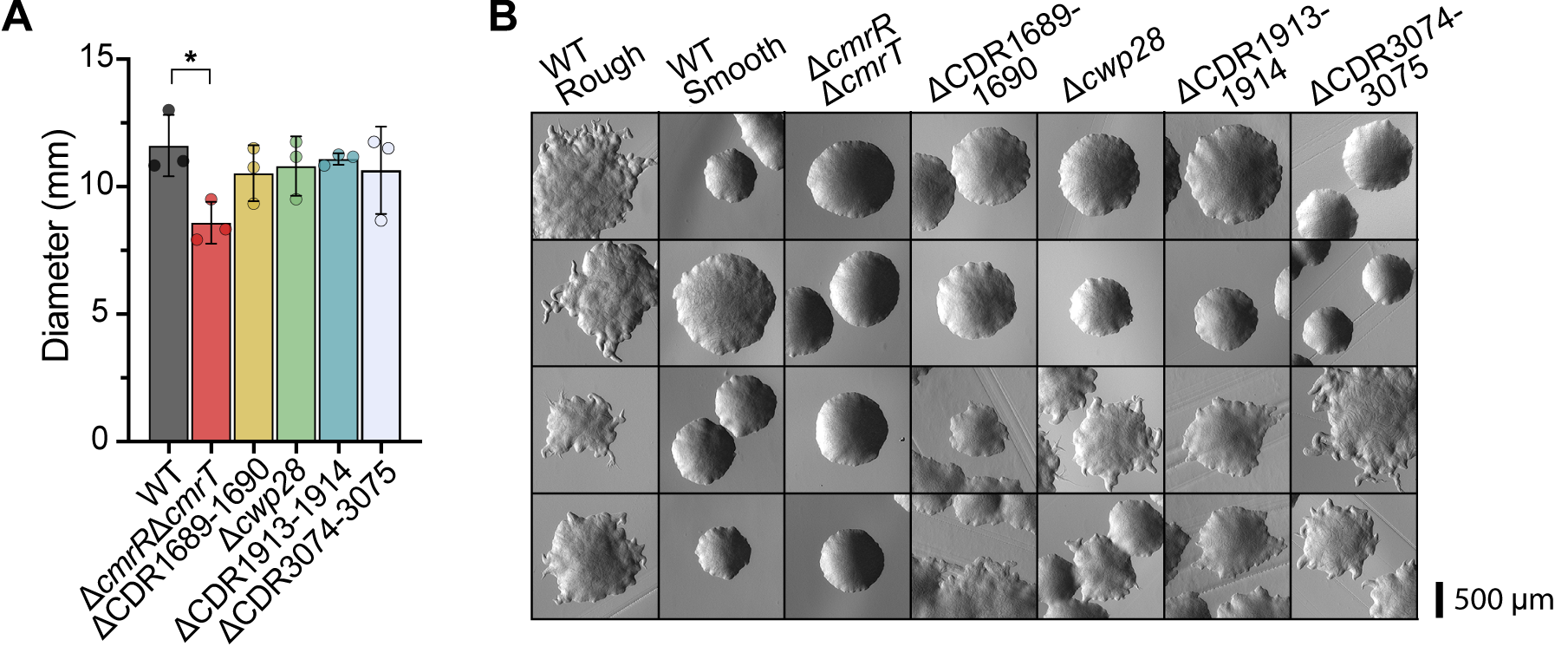

Supplement: S4 Fig — (A) Surface motility assay of strains with CmrR- or CmrT-regulated gene deletions along with wildtype and ΔcmrRΔcmrT controls. Overnight cultures (5 μL) were spotted on BHIS-agar, and surface motility was measured after seven days at 37°C. Shown are means and standard deviation for three biological replicates. *p < 0.05, one-way ANOVA with Dunnett’s post-test. (B) Colony morphology of CmrR- and/or CmrT-regulated gene deletion strains grown on BHIS-agar along with wildtype rough, wildtype smooth, and ΔcmrRΔcmrT controls. Four colonies are shown per strain representing both rough and smooth colony morphologies. Images were taken at 2X magnification after 24 hours at 37°C. Shown are representative images from four biological replicates. (TIF) [file ppat.1013471.s004.tif]

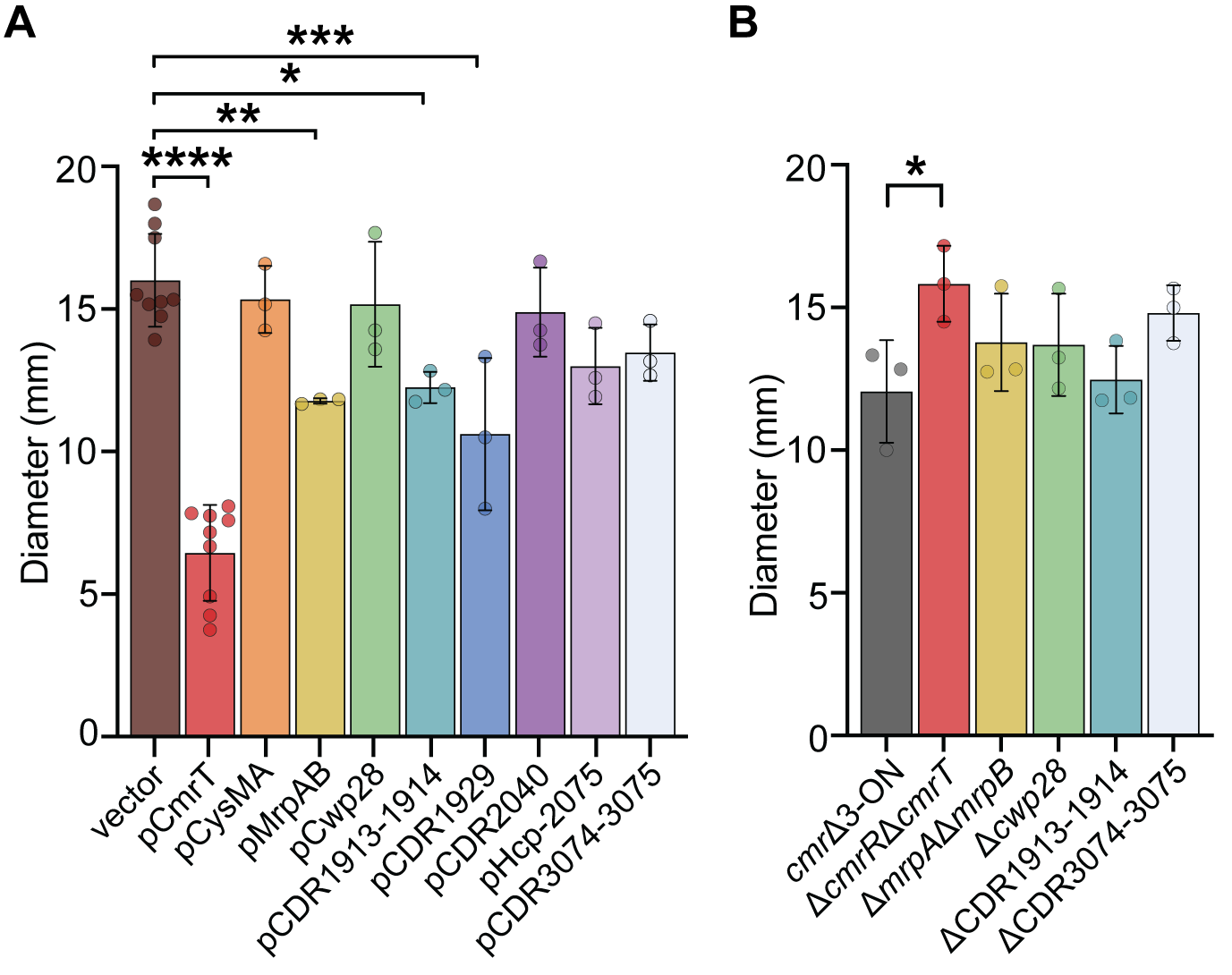

Supplement: S5 Fig — (A-B) Swimming motility was assayed by inoculating a colony of each strain into soft agar (0.5X BHIS-0.3% agar), supplemented with Tm10 and ATc10 in (A). The diameter of motile growth was measured after 48 hours at 37°C. Shown are means and standard deviation for three biological replicates. *p < 0.05, **p < 0.01, ***p < 0.001, ****p < 0.0001, one-way ANOVA with Dunnett’s post-test. (A) ΔcmrT strains ectopically expressing CmrR- or CmrT-regulated genes along with pCmrT and vector controls. (B) Strains with deletions of CmrR- or CmrT-regulated genes along with cmrΔ3-ON and ΔcmrRΔcmrT controls. (TIF) [file ppat.1013471.s005.tif]

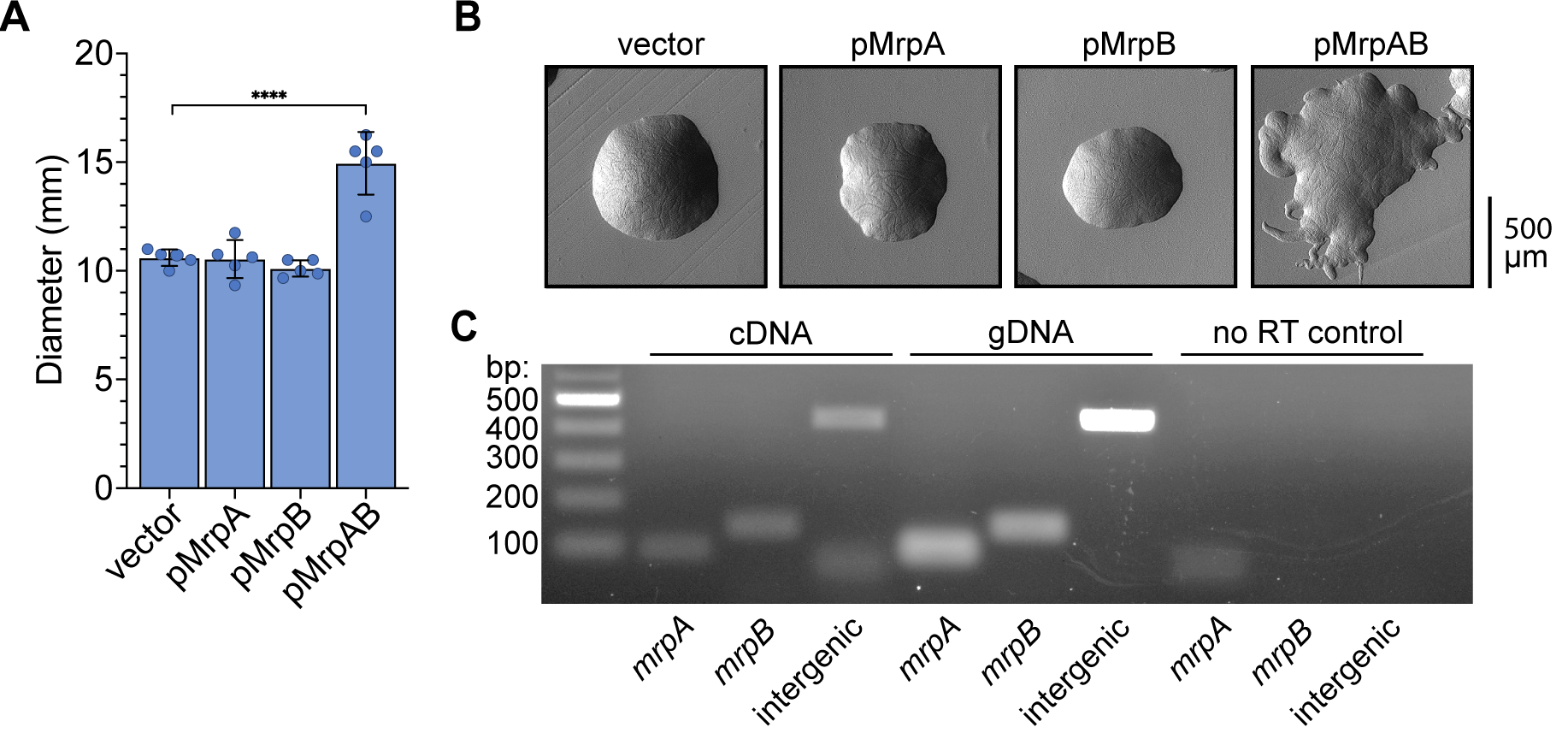

Supplement: S6 Fig — (A) Surface motility assay of ΔcmrT ectopically expressing mrpA, mrpB, or mrpAB along with a vector control. Overnight cultures were spotted on BHIS-Tm10-ATc100-agar, and surface motility was measured after seven days at 37°C. Shown are means and standard deviation for five biological replicates. ****p < 0.0001, one-way ANOVA with Dunnett’s post-test. (B) Colony morphology of ΔcmrT ectopically expressing mrpA, mrpB, or mrpAB along with a vector control. Strains were grown on BHIS-Tm10-ATc100-agar. Images were taken at 2X magnification after 24 hours at 37°C. Shown are representative images from four biological replicates. (C) mrpA and mrpB are co-transcribed. RNA was isolated from wildtype overexpressing cmrT and reverse transcribed to cDNA. Primers were designed to amplify regions within the mrpA and mrpB coding sequence, and a region bridging the intergenic sequence between these genes. Reactions with genomic DNA and no-reverse transcriptase templates were included as positive and negative controls, respectively. (TIF) [file ppat.1013471.s006.tif]

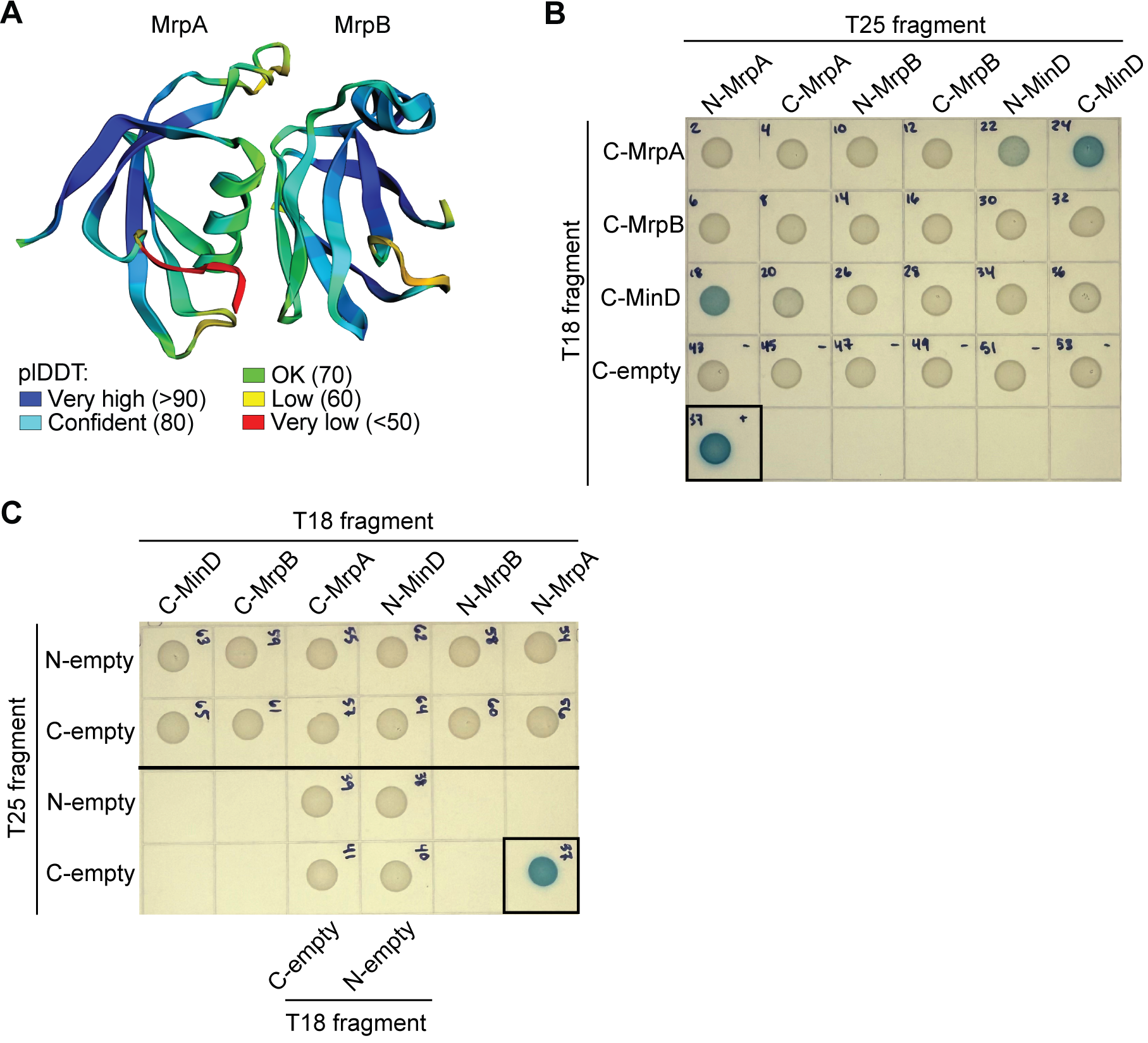

Supplement: S7 Fig — (A) Predicted dimer modeling of MrpA and MrpB by ColabFold, a derivative of AlphaFold2. Colors display the plDDT per-residue confidence metric indicated in the legend (bottom). (B-C) Bacterial two-hybrid results continued from Fig 7. The black square indicates the zip-zip positive control. (B) MrpA, MrpB, and MinD were translationally fused to the C-terminus of the T18 CyaA fragment and tested for interactions with MrpA, MrpB, or MinD fused to the N- or C-termini of the CyaA T25 fragment. A T18 fragment with no translational fusion (“empty”) was included as a negative control. (C) Negative controls for the bacterial two-hybrid assay. The T25 fragment with no translational fusion (“empty”) was tested for interactions with the T18 fragment. (TIF) [file ppat.1013471.s007.tif]
