# Supplementary material for: Phase variation of Clostridioides difficile colony morphology occurs via modulation of cell division
Source: PLoS Pathog. 2025 Dec 1;21(12):e1013471. doi: 10.1371/journal.ppat.1013471 (PMC12694794; doi:10.1371/journal.ppat.1013471)
Supplement: S1 Table — (PDF) [file ppat.1013471.s008.pdf]

Table S1. Strains and plasmids used in this study.

***C. difficile* strains**			
**Lab Notation**	**Strain**	**Description/Usage**	**Reference**
RT273	Wildtype R20291	*Clostridioides difficile* R20291 (GenBank: FN545816.1)	(1)
RT2406	*cmr*Δ3-ON	R20291 with the *cmr* switch locked in the "ON" orientation	(2)
RT2395	*cmr*Δ3-OFF	R20291 with the *cmr* switch locked in the "OFF" orientation	(2)
RT2296	Δ*cmrR*Δ*cmrT*	R20291 with *cmrR* (CDR20291_3128) and *cmrT* (CDR20291_3126) deleted	(3)
RT1615	WT vector, *flg*-ON	R20291 with pRT1611, *flg* phase ON; used for RNA-Seq experiments	(4)
RT2196	WT vector	R20291 with pRT1611	(3)
RT2085	WT pCmrR	R20291 with pRT2073 for inducible expression of *cmrR*	(3)
RT2107	WT pCmrT	R20291 with pRT2106 for inducible expression of *cmrT*	(3)
RT2307	Δ*cmrR*Δ*cmrT* vector	Δ*cmrR*Δ*cmrT* with pRT1611	(5)
RT2308	Δ*cmrR*Δ*cmrT* pCmrR	Δ*cmrR*Δ*cmrT* with pRT2073 for inducible expression of *cmrR*	This work
RT2309	Δ*cmrR*Δ*cmrT* pCmrT	Δ*cmrR*Δ*cmrT* with pRT2106 for inducible expression of *cmrT*	(5)
RT2256	Δ*cmrR*	R20291 with *cmrR* deleted	(3)
RT2257	Δ*cmrT*	R20291 with *cmrT* deleted	(3)
RT2269	Δ*cmrT* vector	Δ*cmrT* with pRT1611	(3)
RT2270	Δ*cmrT* pCmrT	Δ*cmrT* with pRT2106 for inducible expression of *cmrT*	(3)
RT3400	Δ*cmrT* pCysMA	Δ*cmrT* with pRT3393 for inducible expression of CDR20291_1492-1493 (*cysMA*)	This work
RT2981	Δ*cmrT* pCDR1689-1690 (pMrpAB)	Δ*cmrT* with pRT2525 for inducible expression of CDR20291_1689-1690 (*mrpAB*)	This work
RT2982	Δ*cmrT* pCwp28	Δ*cmrT* with pRT2530 for inducible expression of CDR20291_1911 (*cwp28*)	This work
RT2983	Δ*cmrT* pCDR1913-1914	Δ*cmrT* with pRT2969 for inducible expression of CDR20291_1913-1914	This work
RT3389	Δ*cmrT* pCDR1929	Δ*cmrT* with pRT3387 for inducible expression of CDR20291_1929	This work
RT3383	Δ*cmrT* pCDR2040	Δ*cmrT* with pRT3381 for inducible expression of CDR20291_2040	This work
RT3386	Δ*cmrT* pHcp-2075	Δ*cmrT* with pRT3384 for inducible expression of CDR20291_2074 (*hcp*)-2075	This work
RT2985	Δ*cmrT* pCDR3074-3075	Δ*cmrT* with pRT2985 for inducible expression of CDR20291_3074-3075	This work
RT3350	Δ*cmrT* pMrpA	Δ*cmrT* with pRT2960 for inducible expression of CDR20291_1689 (*mrpA*)	This work

RT3351	Δ*cmrT* pMrpB	Δ*cmrT* with pRT2963 for inducible expression of CDR20291_1690 (*mrpB*)	This work
RT3022	ΔCDR1689-1690 (Δ*mrpAB*)	R20291 with CDR20291_1689-1690 (*mrpAB*) deleted	This work
RT2968	Δ*cwp28*	R20291 with CDR20291_1911 (*cwp28*) deleted	This work
RT2972	ΔCDR1913-1914	R20291 with CDR20291_1913-1914 deleted	This work
RT2992	ΔCDR3074-3075	R20291 with CDR20291_3074-3075 deleted	This work
RT2673	WT pVector-FLAG	R20291 with pRT2658 (FLAG-tag vector control)	This work
RT3358	WT pMrpA-FLAG	R20291 with pRT3354 for inducible expression of CDR20291_1689 (*mrpA*) with a translational fusion of a serine-glycine linker and a 3xFLAG-tag	This work
RT3359	WT pMrpB-FLAG	R20291 with pRT3355 for inducible expression of CDR20291_1690 (*mrpB*) with a translational fusion of a serine-glycine linker and a 3xFLAG-tag	This work
RT3362	WT pVector-FLAG; rough isolate	Rough colony isolate of RT2673	This work
RT3363	WT pMrpA-FLAG; rough isolate	Rough colony isolate of RT3358	This work
RT3364	WT pMrpBFLAG; rough isolate	Rough colony isolate of RT3359	This work
***E. coli* Strains**			
**Lab Notation**	**Strain**	**Description/Usage**	**References**
AC472	DH5α	F- φ80*lacZ*ΔM15 Δ(*lacZYA-argF*)U169 *recA1 endA1 hsdR*17(rκ -, mκ+) *phoA supE44 thi*-1 *gyrA96 relA1* λ- *tonA*	Invitrogen, (6)
RT270	HB101(pRK24)	Used for conjugation of plasmids into *C. difficile*	(7)
RT3354	DH5α pMrpA-FLAG	DH5α with pRT3354 for inducible expression of CDR20291_1689 (*mrpA*) with a translational fusion of a serine-glycine linker and a 3xFLAG-tag	This work
RT3355	DH5α pMrpB-FLAG	DH5α with pRT3354 for inducible expression of CDR20291_1690 (*mrpB*) with a translational fusion of a serine-glycine linker and a 3xFLAG-tag	This work
RT2272	BTH101	*E. coli cya-* mutant requiring plasmid-borne expression of *cyaA* to activate the *lac* operon *(F-, cya-99, araD139, galE15, galK16, rpsL1 (Str r), hsdR2, mcrA1, mcrB1)*	Euromedex
**Plasmids**	**Strain**	**Description/Usage**	**References**
pRT709	pRPF185	*E. coli*−*C. difficile* shuttle vector; Expression vector with a tetracycline-inducible promoter (P_{tet}) driving expression of *gusA*	(8)
pRT1611	pRPF185 *gusA*-	Derivative of pRPF185 with *gusA* removed; empty vector control	(4)
pRT2073	pCmrR	Inducible expression vector pRPF185 with *gusA* replaced by *cmrR*	(3)
pRT2106	pCmrT	Inducible expression vector pRPF185 with *gusA* replaced by *cmrT*	(3)

pRT3393	pCDR1492-1493	Inducible expression vector pRPF185 with *gusA* replaced by CDR20291_1492-1493	This work
pRT2525	pCDR1689-1690 (pMrpAB)	Inducible expression vector pRPF185 with *gusA* replaced by CDR20291_1689-1690 (*mrpAB*)	This work
pRT2530	pCwp28	Inducible expression vector pRPF185 with *gusA* replaced by CDR20291_1911 (*cwp28*)	This work
pRT2969	pCDR1913-1914	Inducible expression vector pRPF185 with *gusA* replaced by CDR20291_1913-1914	This work
pRT3387	pCDR1929	Inducible expression vector pRPF185 with *gusA* replaced by CDR20291_1929	This work
pRT3381	pCDR2040	Inducible expression vector pRPF185 with *gusA* replaced by CDR20291_2040	This work
pRT3384	pHcp-2075	Inducible expression vector pRPF185 with *gusA* replaced by CDR20291_2074 (*hcp*)-2075	This work
pRT2526	pCDR3074-3075	Inducible expression vector pRPF185 with *gusA* replaced by CDR20291_3074-3075	This work
pRT2960	pMrpA	Inducible expression vector pRPF185 with *gusA* replaced by CDR20291_1689 (*mrpA*)	This work
pRT2963	pMrpB	Inducible expression vector pRPF185 with *gusA* replaced by CDR20291_1690 (*mrpB*)	This work
pRT2460	pMSR0	*E. coli−C. difficile* shuttle vector for toxin/antitoxin mediated allelic exchange	(9)
pRT2840	pMSR0::ΔCDR1689-1690	Toxin/antitoxin mediated allelic exchange vector with homology arms designed for deletion of CDR20291_1689-1690 (*mrpAB*)	This work
pRT2967	pMSR0::Δ*cwp28*	Toxin/antitoxin mediated allelic exchange vector with homology arms designed for deletion of CDR20291_1911 (*cwp28*)	This work
pRT2971	pMSR0::ΔCDR1913-1914	Toxin/antitoxin mediated allelic exchange vector with homology arms designed for deletion of CDR20291_1913-1914	This work
pRT2991	pMSR0::ΔCDR3074-3075	Toxin/antitoxin mediated allelic exchange vector with homology arms designed for deletion of CDR20291_3074-3075	This work
pRT2658	pRT1611::3xFLAG	pRT1611 with 3xFLAG-tag sequence for translational fusions	This work
pRT3354	pCDR1689-3xFLAG	Inducible expression vector pPRF185 with *gusA* replaced by CDR20291_1689 translationally fused to a serine-glycine linker and 3xFLAG-tag	This work
pRT3355	pCDR1690-3xFLAG	Inducible expression vector pPRF185 with *gusA* replaced by CDR20291_1690 translationally fused to a serine-glycine linker and 3xFLAG-tag	This work
pRT3372	pUC-GW-Kan::CDR1689-Opt	CDR20291_1689 (*mrpA)* codon optimized sequence for translation in *E. coli*	This work
pRT3373	pUC-GW-Kan::CDR1690-Opt	CDR20291_1690 (*mrpB)* codon optimized sequence for translation in *E. coli*	This work
pRT3374	pUC-GW-Kan::CDR0987-Opt	CDR20291_0987 (*minD/divIVB)* codon optimized sequence for translation in *E. coli*	This work

pRT2278	pKT25-zip	T25 fragment of CyaA fused to a GCN4 leucine zipper; used with pUT18C-zip as a Cya+ positive control	Euromedex, (10)
pRT2279	pUT18C-zip	T18 fragment of CyaA fused to a GCN4 leucine zipper; used with pKT25-zip as a Cya+ positive control	Euromedex, (10)
pRT2274	pKNT25	T25 fragment of CyaA with multiple cloning site at 5' end; used for N-terminal fusions of the protein of interest to T25	Euromedex, (10)
pRT2275	pKT25	T25 fragment of CyaA with multiple cloning site at 3' end; used for C-terminal fusions of the protein of interest to T25	Euromedex, (10)
pRT2276	pUT18	T18 fragment of CyaA with multiple cloning site at 5' end; used for N-terminal fusions of the protein of interest to T18	Euromedex, (10)
pRT2277	pUT18C	T18 fragment of CyaA with multiple cloning site at 3' end; used for C-terminal fusions of the protein of interest to T18	Euromedex, (10)
pRT3410	pKNT25::*mrpA*	CDR20291_1689 (*mrpA)* N-terminally fused to the T25 fragment of CyaA	This work
pRT3411	pKNT25::*mrpB*	CDR20291_1690 (*mrpB*) N-terminally fused to the T25 fragment of CyaA	This work
pRT3412	pKNT25::*minD*	CDR20291_0987 (*minD)* N-terminally fused to the T25 fragment of CyaA	This work
pRT3413	pKT25::*mrpA*	CDR20291_1689 (*mrpA*) C-terminally fused to the T25 fragment of CyaA	This work
pRT3414	pKT25::*mrpB*	CDR20291_1690 (*mrpB*) C-terminally fused to the T25 fragment of CyaA	This work
pRT3415	pKT25::*minD*	CDR20291_0987 (*minD*) C-terminally fused to the T25 fragment of CyaA	This work
pRT3416	pUT18::*mrpA*	CDR20291_1689 (*mrpA*) N-terminally fused to the T18 fragment of CyaA	This work
pRT3417	pUT18::*mrpB*	CDR20291_1690 (*mrpB*) N-terminally fused to the T18 fragment of CyaA	This work
pRT3418	pUT18::*minD*	CDR20291_0987 (*minD*) N-terminally fused to the T18 fragment of CyaA	This work
pRT3419	pUT18C::*mrpA*	CDR20291_1689 (*mrpA*) C-terminally fused to the T18 fragment of CyaA	This work
pRT3420	pUT18C::*mrpB*	CDR20291_1690 (*mrpB*) C-terminally fused to the T18 fragment of CyaA	This work
pRT3421	pUT18C::*minD*	CDR20291_0987 (*minD*) C-terminally fused to the T18 fragment of CyaA	This work

References:

1. Stabler RA, He M, Dawson L, Martin M, Valiente E, Corton C, et al. Comparative genome and phenotypic analysis of *Clostridium difficile* 027 strains provides insight into the evolution of a hypervirulent bacterium. Genome Biol. 2009;10(9):1–15. doi:10.1186/gb-2009-10-9-r102

2. Garrett EM, Mehra A, Sekulovic O, Tamayo R. Multiple Regulatory Mechanisms Control the Production of CmrRST, an Atypical Signal Transduction System in *Clostridioides difficile*. mBio. 2022;13(1):e02969-21. doi:10.1128/mbio.02969-21

3. Garrett EM, Sekulovic O, Wetzel D, Jones JB, Edwards AN, Vargas-Cuebas G, et al. Phase variation of a signal transduction system controls *Clostridioides difficile* colony morphology, motility, and virulence. PLoS Biol. 2019;17(10):e3000379. doi:10.1371/journal.pbio.3000379

4. Anjuwon-Foster BR, Tamayo R. A genetic switch controls the production of flagella and toxins in *Clostridium difficile*. PLoS Genet. 2017;13(3):e1006701. doi:10.1371/journal.pgen.1006701

5. Williams CL, Mehra A, Harrison EK, Thomas CG, Palmer JR, Cooper DK, et al. Characterization of two co-regulated response regulators in *Clostridioides difficile*. J Bacteriol. 2025;207(10). doi:10.1128/jb.00177-25

6. Hanahan D. Studies on transformation of *Escherichia coli* with plasmids. J Mol Biol. 1983;166(4):557–80. doi:10.1016/S0022-2836(83)80284-8

7. McBride SM, Sonenshein AL. Identification of a Genetic Locus Responsible for Antimicrobial Peptide Resistance in *Clostridium difficile*. Infect Immun. 2011;79(1):167–76. doi:10.1128/IAI.00731-10

8. Fagan RP, Fairweather NF. *Clostridium difficile* Has Two Parallel and Essential Sec Secretion Systems. Journal of Biological Chemistry. 2011;286(31):27483–93. doi:10.1074/jbc.M111.263889

9. Peltier J, Hamiot A, Garneau JR, Boudry P, Maikova A, Hajnsdorf E, et al. Type I toxin-antitoxin systems contribute to the maintenance of mobile genetic elements in *Clostridioides difficile*. Commun Biol. 2020;3(1):718. doi:10.1038/s42003-020-01448-5

10. Karimova G, Pidoux J, Ullmann A, Ladant D. A bacterial two-hybrid system based on a reconstituted signal transduction pathway. Proceedings of the National Academy of Sciences. 1998;95(10):5752–6. doi:10.1073/pnas.95.10.5752
