# Supplementary material for: Phase variation of Clostridioides difficile colony morphology occurs via modulation of cell division
Source: PLoS Pathog. 2025 Dec 1;21(12):e1013471. doi: 10.1371/journal.ppat.1013471 (PMC12694794; doi:10.1371/journal.ppat.1013471)
Supplement: S2 Table — (PDF) [file ppat.1013471.s009.pdf]

Table S2. Oligonucleotides used in this study.

Primer	Name	Sequence
**qRT-PCR**		
R850	rpoC-qF	CTAGCTGCTCCTATGTCTCACATC
R851	rpoC-qR	CCAGTCTCTCCTGGATCAACTA
R3224	CDR1689-qF	TTTATGTTACAGTCTATAAAGATGAAAGTTCTG
R3225	CDR1689-qR	GCAACAACTTCTCCATCCCATAC
R3228	CDR1911-qF	CTATACCAACTGAGACATCTGCAAG
R3229	CDR1911-qR	ATTCTGTCATTTCCCTTAATTCTTACAAC
R2820	CDR1914-qF	TTGGCAAATCTAGCATCAATTTTAAAAC
R2821	CDR1914-qR	CTCTCAACTTCTGCCCATATTCC
R3242	CDR3075-qF	CATAATTCTTCTAAACCTATATTGGGAACTATC
R3243	CDR3075-qR	TGGCAATAATGCATAAATGGTAAGTG
R2826	CDR2040-qF	CAGTGAAACTCAAAAGGTTGTTAAGC
R2827	CDR2040-qR	CATCTTTGAAATCCATTTTCTCCATTACC
R3918	CDR2075-qF	TGAGGGAAAGTGTAATGGTTGTG
R3919	CDR2075-qR	ATGAAATCGCATTTGTTGGACAAG
R2539	CDR3127-qF	GATAGATGACTGGGAG
R2540	CDR3127-qR	CGATAAGTAGCATTCCC
**Gene overexpression**		
R3888	pCDR1492-1493-F	cgtagcgttaacagatctgagctcTGGACATATAGGAGGAAATAG
R3889	pCDR1492-1493-R	gttttattaaaacttataggatccTCCTATATATAATATACATTATCTAATTCATC
R2755	pCDR1689-1690-F	cgtagcgttaacagatctgagctcGAGATTGGAGGAATTGCTTTG
R2756	pCDR1689-1690-R	gttttattaaaacttataggatccTCCAATATTCTAAAATTAATTTTG
R2832	pCDR1689-R	gttttattaaaacttataggatccCTATATAATTAGACTATTATCCAGTTTTAACC
R2833	pCDR1690-F	cgtagcgttaacagatctgagctcCGATACTGGAGGAGTAATTCAATGG
R2834	pCDR1690-R	gttttattaaaacttataggatccCTAAAATTAATTTTGAATTGTATTGATTGAAAAG
R2757	pCDR1911-F	cgtagcgttaacagatctgagctcGAGGGGGACAATTTTATTATGAAATTC
R2758	pCDR1911-R	gttttattaaaacttataggatccCATCTTTCCTTAAATAAAATATATGATTTAC
R3023	pCDR1913-1914-F	cgtagcgttaacagatctgagctcATAACTTAATATTTGGCAAGAGGAG
R3024	pCDR1913-1914-R	gttttattaaaacttataggatccTATGTCTTCTTGCATTTCGTCT
R3890	pCDR1929-F	cgtagcgttaacagatctgagctcAATTAAGTAAATGGAGGAATTG
R3891	pCDR1929-R	gttttattaaaacttataggatccACTATTTAGCTGGCATCTG
R3698	pCDR2040-F	cgtagcgttaacagatctgagctcACAAATGAGGTAACAATAATG
R3699	pCDR2040-R	gttttattaaaacttataggatccTAAAATGACTCAATGTATAGACTG
R3700	pCDR2074-2075-F	cgtagcgttaacagatctgagctcAATTTTATGAGGTGATATAAATG
R3701	pCDR2074-2075-R	gttttattaaaacttataggatccTTATCCTAAAATCTTCTTCATATC
R2763	pCDR3074-3075-F	cgtagcgttaacagatctgagctcCTTATAAAAGGAGAGAGATGGATG
R2764	pCDR3075-3075-R	gttttattaaaacttataggatccGTATACAACTTGAAGTTAGCTC
**Gene deletions**		
R2991	CDR1689-F1	cattgatttctttcagtttcggatccGATTGTGTGACTAAGTCACAGAAAG
R2999	CDR1689-1690-R1	gtacaaaaagtaagaatatccaatattctaCAAAGCAATTCCTCCAATCTC
R3000	CDR1689-1690-F2	gagattggaggaattgctttgTAGAATATTGGATATTCTTACTTTTTGTAC
R2998	CDR1690-R2	gacgtcgactctagaggatccGGTGTAAAATTTGAAGATATTGAAGCTC
R3001	CDR3074-F1	cattgatttctttcagtttcggatccGACAATTAGTGGAAGTATACTTATTGCTC
R3009	CDR3074-3075-R1	ctattatagatttcttatattcaataactggtctacCATCCATCTCTCTCCTTTTATAAGTTTTAC
R3010	CDR3074-3075-F2	gtaaaacttataaaaggagagagatggatgGTAGACCAGTTATTGAATATAAGAAAATCT ATAATAG
R3008	CDR3075-R2	gacgtcgactctagaggatccCACTTCTATTTGAAGTGCCTATCTACTC
R3015	CDR1911-F1	ttcggatcctctagagtcgacGAACTATCTTTTTATTAGCAGCTC

R3016	CDR1911-R1	caattacatctattcattttcaatGAATTTCATAATAAAATTGTCC
R3017	CDR1911-F2	ggacaattttattatgaaattcATTGAAAATGAATAGATGTAATTG
R3018	CDR1911-R2	atgtctgcaggcctcgagCAAGTGTCTTACCATAAGAATCTAC
R3019	CDR1914-F1	cattgatttctttcagtttcggatccACAGACTAAACACGTAGAGTACC
R3020	CDR1914-R1	gtcttcttgcatttcgtcttttatatttaAGATATCATCTTAAGCCCTCCTCTTG
R3021	CDR1913-F2	caagaggagggcttaagatgatatctTAAATATAAAAGACGAAATGCAAGAAGAC
R3022	CDR1913-R2	gacgtcgactctagaggatccCCTAAACTAATAGGACGGAGATTATC
**OS-qPCR**		
R2273	rpoA-qF	TCATTACCAGGTGTAGCAGTGAATGC
R2274	rpoA-qR	GATAGAGCATGGTCCTTGAGCTTCT
R2270	cmr-ON	GGAGATATATGGAGTTAGTGGTGCAA
R2271	cmr-common	CTAGCCAATAGACAAGTTTCTAGAAAAATA
R2272	cmr-OFF	GAACAATTCTTGAATATTGTATTGAACATTAAGA
**CDR1689-1690 co-transcription**		
R3226	CDR1690-qF	AGGTAAATTTAGAGAAACAAGCCAATG
R3227	CDR1690-qR	CCATGTTCCAGTATTAGGAACTCTC
**FLAG-tag translational fusions**		
R3595	pCDR1689-FLAG-F	cgttaacagatctgagctcAGAGATTGGAGGAATTGCTATGTTTATAG
R3596	oCDR1689-FLAG-R	cactagaacccccctcgagATCCAGTTTTAACCTTATTTACTACAATCC
R3597	pCDR1690-FLAG-F	cgttaacagatctgagctcATACTGGAGGAGTAATTCAATG
R3598	pCDR1690-FLAG-R	cactagaacccccctcgagAATTTTGAATTGTATTGATTGAAAAG
**Bacterial two-hybrid plasmid construction**		
R3961	pUT18-1689-F	caagcttgcatgcctgcaggATGTTTATTGATGAAGAACTGG
R3962	pUT18-1689-R	ctgaattcgagctcggtacccgGCCGGTTTTCACTTTG
R3963	pUT18C-1689-F	cagtggaacgccactgcaggATGTTTATTGATGAAGAACTGG
R3964	pUT18C-1689-R	gatgaattcgagctcggtacccgGCCGGTTTTCACTTTG
R3965	pKT25-1689-F	gcacgcggcgggctgcagggATGTTTATTGATGAAGAACTGG
R3966	pKT25-1689-R	gtgaattcttacttacttaggtacccgGCCGGTTTTCACTTTG
R3967	pKNT25-1689-F	caagcttgcatgcctgcaggATGTTTATTGATGAAGAACTGG
R3968	pKNT25-1689-R	ttgaattcgagctcggtacccgGCCGGTTTTCACTTTG
R3969	pUT18-1690-F	caagcttgcatgcctgcaggATGGAATATAGCTATAGCAAAATG
R3970	pUT18-1690-R	ctgaattcgagctcggtacccgGTTCTGAATGGTGTTAATGC
R3971	pUT18C-1690-F	cagtggaacgccactgcaggATGGAATATAGCTATAGCAAAATG
R3972	pUT18C-1690-R	gatgaattcgagctcggtacccgGTTCTGAATGGTGTTAATGC
R3973	pKT25-1690-F	gcacgcggcgggctgcagggATGGAATATAGCTATAGCAAAATG
R3974	pKT25-1690-R	gtgaattcttacttacttaggtacccgGTTCTGAATGGTGTTAATGC
R3975	pKNT25-1690-F	caagcttgcatgcctgcaggATGGAATATAGCTATAGCAAAATG
R3976	pKNT25-1690-R	ttgaattcgagctcggtacccgGTTCTGAATGGTGTTAATGC
R3977	pUT18-0987-F	caagcttgcatgcctgcaggATGAGCGAAGTGATTGTG
R3978	pUT18-0987-R	ctgaattcgagctcggtacccgTTTCGCCATGCTAAAC
R3979	pUT18C-0987-F	cagtggaacgccactgcaggATGAGCGAAGTGATTGTG
R3980	pUT18C-0987-R	gatgaattcgagctcggtacccgTTTCGCCATGCTAAAC
R3981	pKT25-0987-F	gcacgcggcgggctgcagggATGAGCGAAGTGATTGTG
R3982	pKT25-0987-R	gtgaattcttacttacttaggtacccgTTTCGCCATGCTAAAC
R3983	pKNT25-0987-F	caagcttgcatgcctgcaggATGAGCGAAGTGATTGTG
R3984	pKNT25-0987-R	ttgaattcgagctcggtacccgTTTCGCCATGCTAAAC

[a]Underlined text = Restriction enzyme sites
[b]Lowercase text = Gibson homology arms
[c]Red text = Nucleotide(s) required to keep translation in frame
[d]Blue text = Nucleotide changed to create an ATG start codon in place of a TTG start codon
