# Supplementary material for: Phase variation of Clostridioides difficile colony morphology occurs via modulation of cell division
Source: PLoS Pathog. 2025 Dec 1;21(12):e1013471. doi: 10.1371/journal.ppat.1013471 (PMC12694794; doi:10.1371/journal.ppat.1013471)
Supplement: S1 Text — (PDF) [file ppat.1013471.s016.pdf]

SUPPLEMENTAL MATERIALS AND METHODS

Strain and Plasmid Construction

The *C. difficile* R20291 genome (NCBI Accession No. FN545816) was used as a template for all constructs. Anhydrotetracycline (ATc)-inducible expression strains were made by cloning genes of interest into a derivative of pRPF185 in which *gusA* was excised by SacI/BamHI (pRT2611) (1). Genes were PCR amplified with primers indicated in Table S2; primers are labeled as pCDRXXXX-F or -R (forward and reverse primers, respectively), with XXXX as a placeholder for the gene locus that was amplified. The column-purified PCR product and digested vector were then joined by Gibson assembly (NEB).

FLAG-tagged translational fusions were similarly made by Gibson assembly of a 3xFLAG-tag gene block (IDT; sequence available upon request) between the SacI/BamHI sites of pRT2611 (pRPF185 *gusA*-) resulting in plasmid pRT2658. A SphI site, added before the 3xFLAG sequence, was used to clone in *mrpA* (CDR1689) or *mrpB* (CDR1690) followed by a serine-glycine linker (5'-GGSSGGGGSGGGGSSG-3'). Primers R3595/R3596 or R3597/R3598 were used to amplify *mrpA* or *mrpB*, respectively.

Markerless gene deletions were generated using the pMSR0 toxin/antitoxin allelic exchange vector (2). For genes of interest, regions of ~1,000 base pairs up- and downstream the gene were PCR amplified using primers in Table S2; CDRXXXX-F1 and -R1 were used to amplify the upstream region, and CDRXXXX-F2 and -R2 were used to amplify the downstream region. The PCR-purified products were inserted into digested pMSR0 (BamHI for Δ*mrpAB*, ΔCDR1913-1914, and ΔCDR3074-3075, or SalI/XhoI for Δ*cwp28*) using Gibson assembly. These plasmids were then introduced into *C. difficile* using conjugation with *E. coli* HB101(pRK24) as the donor, and allelic exchange was done as previously described (2).

Plasmids used for bacterial two-hybrid (BACTH) were created using *mrpA*, *mrpB*, and *minD* (CDR0987) sequences that were codon-optimized for translation in *E. coli* (Genewiz;

sequences available upon request). Each codon-optimized sequence was amplified using plasmid-specific primers listed in Table S2 to maintain in-frame translation. Amplified products were column purified and cloned into four BACTH plasmids: pKT25 and pKNT25 (the T25 fragment of CyaA for C- and N-terminal translational fusions, respectively), and pUT18C and pUT18 (the T18 fragment of CyaA for C- and N-terminal translational fusions, respectively) (Euromedex) (3, 4). Vectors and inserts were digested with PstI and KpnI and ligated together.

Motility assays

For surface motility assays, overnight cultures (5 µL) of each strain were spotted on 30 mL BHIS 1.5%-agar plates without 0.1% cysteine supplementation. Every plate contained the relevant controls. For overexpression strains, the agar medium was supplemented with Tm10 and ATc (0-100 ng/mL, as indicated). Plates were made two days before use and reduced in the anaerobic chamber overnight one day before use. After allowing spots to dry, plates were loosely wrapped in plastic to minimize desiccation during extended incubation at 37°C. Surface motility was measured after seven days of growth by measuring the widest diameter of each spot and the perpendicular diameter then calculating the average as described previously (5).

Swimming motility plates were prepared with 30 mL of 0.5X BHIS-0.3% agar and supplemented with Tm10 and ATc 0-100 ng/mL as indicated. Colonies of *C. difficile* were inoculated into the agar, then the plates were incubated agar-side-down at 37°C. Diameters were measured after 48 hours by averaging two perpendicular measurements per spot (5–7).

Co-transcription of *mrpA* and *mrpB*

RNA and genomic DNA was isolated from a WT pCmrT strain as described previously (8). RNA was treated with DNase then reverse transcribed into cDNA as described in the qRT-PCR methods. *mrpA*, *mrpB*, and the length spanning *mrpA*-*mrpB* were amplified from DNA, cDNA, and a no reverse transcriptase control templates using primers R3224/R3225 (*mrpA*),

R32226/R3227 (*mrpB*), R3224/R3227 (*mrpA-mrpB*). Products were run on a 1.2% agarose gel and imaged on a SynGene G:Box Chemi XX6.

Western blot

For *E. coli* DH5α strains bearing pMrpA-FLAG or pMrpB-FLAG, overnight cultures were diluted 1:50 in LB-Cm10 and induced with ATc 0, 50, 100, 250, or 500 ng/mL. At OD600 ~1, samples were mixed in equal parts with 2x Laemmli buffer (125 mM Tris HCl, 20% glycerol, 4% SDS, 10% β-mercaptoethanol, 0.02 mg/mL bromophenol blue) and boiled at 100°C for 10 minutes. Samples were separated on 4-15% TGX gels (BioRad 4561086) in running buffer consisting of 25 mM Tris, 192 mM glycine, 0.1% SDS. Proteins were transferred to a nitrocellulose membrane by electrophoresis in transfer buffer (25 mM Tris, 192 mM glycine, 10% methanol). After blocking in 5% powdered milk, the membrane was probed with a 1:10,000 dilution of mouse anti-FLAG antibodies (Sigma F1804), then a 1:10,000 dilution of goat anti-mouse IgG antibody (IR800, LI-COR 926-32210). Membranes were visualized using a LI-COR Odyssey DLx imager.

LC-MS/MS

Immunoprecipitated samples, prepared in triplicate, were subjected to on-bead trypsin digestion, as previously described (9). After the last wash buffer step, 50 µL of 50 mM ammonium bicarbonate (pH 8) containing 1 µg trypsin (Promega) was added to beads overnight at 37ºC with shaking. The next day, 1 µg of trypsin was added then incubated for an additional 3 hours at 37°C with shaking. Supernatants from pelleted beads were transferred, then beads were washed twice with 100 µL LC/MS grade water. These rinses were combined with the original supernatant, then acidified to 0.7% trifluoracetic acid. Peptides were desalted with peptide desalting spin columns (Thermo) and dried via vacuum centrifugation. Peptide samples were stored at -80°C until further analysis.

Each sample was analyzed by LC-MS/MS using an Easy nLC 1200 coupled to a QExactive HF (Thermo Scientific). Samples were injected onto an IonOpticks Aurora Elite TS C18 column (75 μm id × 15 cm, 1.7 μm particle size) and separated over a 120 min method. The gradient for separation consisted of a step gradient from 5 to 36 to 48% mobile phase B at a 250 nL/min flow rate, where mobile phase A was 0.1% formic acid in water and mobile phase B consisted of 0.1% formic acid in 80% ACN. The QExactive HF was operated in data-dependent mode where the 15 most intense precursors were selected for subsequent HCD fragmentation. Resolution for the precursor scan (m/z 350–1700) was set to 60,000 with a target value of 3×10^6 ions, 100ms inject time. MS/MS scans resolution was set to 15,000 with a target value of 1×10^5 ions, 75ms inject time. The normalized collision energy was set to 27% for HCD, with an isolation window of 1.6 m/z. Peptide match was set to preferred, and precursors with unknown charge or a charge state of 1 and ≥ 8 were excluded.

Raw data were processed using the MaxQuant software suite (version 1.6.15.0) for peptide/protein identification and label-free quantitation (10). Data were searched against a Uniprot *Clostridium difficile* R20291 database (downloaded 10/2024, containing 3,506 sequences), appended with MaxQuant's common contaminants database (245 sequences), using the integrated Andromeda search engine. A maximum of two missed tryptic cleavages were allowed. The variable modifications specified were: N-terminal acetylation and oxidation of Met. Label-free quantitation (LFQ) was enabled. Results were filtered to 1% FDR at the unique peptide level and grouped into proteins within MaxQuant. Match between runs was enabled. Data filtering and statistical analysis was performed in Perseus software (version 1.6.14.0) (11). LFQ intensities were $\log_2$ transformed and filtered for valid values in minimum 2 / 3 replicates in either of the experimental IPs. Missing values were imputed from a normal distribution with downshift of 1.8 and width of 0.3 the standard deviation. Student's T-tests with FDR-permutation were computed and proteins with $\log_2$ fold change ≥ 1 and a corrected p-value (q-value) < 0.05 were considered significant.

Scanning electron microscopy

Strains were grown in TY-Tm10 broth supplemented with ATc 2 ng/mL (vector or pCmrT) or 100 ng/mL (vector and pMrpAB). Overnight cultures (5 µL) were inoculated on BHIS-Tm10-agar supplemented with ATc 2 or 100 ng/mL, as indicated above. After four days, bacterial colonies on agar plates were vapor-fixed upside down in a sealed plastic bag using 8% paraformaldehyde/12.5% glutaraldehyde in deionized water overnight, pH 7.4, followed by excising the colony, transferring to a humidified 12-well plate and applying a coating of 2% paraformaldehyde/2.5% glutaraldehyde in 0.15M sodium phosphate buffer to the colony and held at 4°C overnight. Colonies were gently rinsed with deionized water for 5 min and dehydrated slowly by submersion through an ascending series of ethanol (30%, 50%, 75%, 100%, 100% 100%) and held in 100% ethanol overnight at 4°C. Following a 100% ethanol exchange and brief hold, colonies were transferred into fresh 100% ethanol to a Samdri-795 critical point dryer and dried using liquid carbon dioxide as the transitional solvent (Tousimis Research Corporation, Rockville, MD). Samples were then transferred to a house air vacuum chamber overnight. The agar sections were then mounted to 13 mm coverslips and attached with double-sided carbon adhesive tabs to 13 mm diameter aluminum stubs and sputter coated with 10 nm of a 60:40 gold/palladium alloy using a Cressington 208HR Sputter Coater (Ted Pella Inc, Redding CA). Images were obtained using a Zeiss Supra 25 FESEM operating at 5 kV using an InLens detector, 20 µm aperture, and approximate working distances of 8 mm (Carl Zeiss Microscopy, LLC, Peabody, MA).

REFERENCES

1. Fagan RP, Fairweather NF. *Clostridium difficile* Has Two Parallel and Essential Sec Secretion Systems. Journal of Biological Chemistry. 2011;286(31):27483–93. doi:10.1074/jbc.M111.263889

2. Peltier J, Hamiot A, Garneau JR, Boudry P, Maikova A, Hajnsdorf E, et al. Type I toxin-antitoxin systems contribute to the maintenance of mobile genetic elements in *Clostridioides difficile*. Commun Biol. 2020;3(1):718. doi:10.1038/s42003-020-01448-5

3. Karimova G, Pidoux J, Ullmann A, Ladant D. A bacterial two-hybrid system based on a reconstituted signal transduction pathway. Proceedings of the National Academy of Sciences. 1998;95(10):5752–6. doi:10.1073/pnas.95.10.5752

4. Olson MG, Goldammer M, Gauliard E, Ladant D, Ouellette SP. A Bacterial Adenylate Cyclase-Based Two-Hybrid System Compatible with Gateway® Cloning. In: Methods in molecular biology (Clifton, NJ). Humana Press Inc.; 2018. p. 75–96. doi:10.1007/978-1-4939-7871-7_6

5. Garrett EM, Sekulovic O, Wetzel D, Jones JB, Edwards AN, Vargas-Cuebas G, et al. Phase variation of a signal transduction system controls *Clostridioides difficile* colony morphology, motility, and virulence. PLoS Biol. 2019;17(10):e3000379. doi:10.1371/journal.pbio.3000379

6. Purcell EB, McKee RW, McBride SM, Waters CM, Tamayo R. Cyclic Diguanylate Inversely Regulates Motility and Aggregation in *Clostridium difficile*. J Bacteriol. 2012;194(13):3307–16. doi:10.1128/JB.00100-12

7. Anjuwon-Foster BR, Maldonado-Vazquez N, Tamayo R. Characterization of Flagellum and Toxin Phase Variation in *Clostridioides difficile* Ribotype 012 Isolates. J Bacteriol. 2018;200(14):1–15. doi:10.1128/JB.00056-18

8. Bouillaut L, McBride SM, Sorg JA. Genetic Manipulation of *Clostridium difficile*. Curr Protoc Microbiol. 2011;20:9A.2.1-9A.2.17. doi:10.1002/9780471729259.mc09a02s20

9. Parsons VA, Vadlamudi S, Voos KM, Rohy AE, Moxley AH, Cannon ME, et al. TBC1D30 regulates proinsulin and insulin secretion and is the target of a genomic association signal for proinsulin. Diabetologia. 2025;68(6):1169–83. doi:10.1007/s00125-025-06391-w

10. Tyanova S, Temu T, Cox J. The MaxQuant computational platform for mass spectrometry-based shotgun proteomics. Nature Protocols 2016 11:12. 2016;11(12):2301–19. doi:10.1038/nprot.2016.136

11. Tyanova S, Cox J. Perseus: A Bioinformatics Platform for Integrative Analysis of Proteomics Data in Cancer Research. In: Methods in Molecular Biology. Humana Press Inc.; 2018. p. 133–48. doi:10.1007/978-1-4939-7493-1_7
